# Manifestations of the oral mucosa and salivary glands in irritable bowel syndrome and microscopic colitis – A systematic review

**DOI:** 10.2340/aos.v84.43870

**Published:** 2025-06-11

**Authors:** Hanna Göthlin, Bengt Hasséus, Klas Sjöberg, Maria Bankvall

**Affiliations:** a Department of Oral Medicine & Pathology, Institute of Odontology, The Sahlgrenska Academy, University of Gothenburg, Gothenburg, Sweden; b Department of Clinical Sciences, Malmö, Lund University, Malmö, Sweden; c Department of Gastroenterology and Nutrition, Skåne University Hospital, Malmö, Sweden; d Department of Dental Medicine, Karolinska Institute, Huddinge, Sweden; e Department of Odontology and Oral Sciences, School of Health and Welfare, University of Jönköping, Sweden

**Keywords:** Gastrointestinal disease, irritable bowel syndrome, microscopic colitis, oral cavity, oral mucosal disease

## Abstract

**Objective:**

There is a well-established association of oral manifestations in the non-infectious chronic diarrhoeal conditions namely, Morbus Crohn, ulcerative colitis, and coeliac disease. Such a connection may exist also for the remaining non-infectious chronic diarrhoeal conditions, that is irritable bowel syndrome (IBS) and microscopic colitis (MC).

**Materials and methods:**

A systematic search was performed in Scopus and PubMed, rendering a total of 710 articles to be screened. All articles were screened independently and assessed for eligibility reporting comorbidity between either IBS or MC, and oral symptoms/disease. Quality assessment and data extraction were performed.

**Results:**

In all, 17 articles were included. Sjögren’s syndrome (SS) in patients with IBS ranged from 3% to 33% and for IBS in SS between 29% to 62%. Dry mouth, bad breath, and foul taste were overrepresented in these patients. The occurrence of SS in patients with MC ranged from 2% to 9%, and for MC in SS from 1% to 2%.

**Conclusions:**

An association between SS and IBS and MC, respectively, is plausible. Few articles have explored other oral manifestations. Therefore, no specific conclusions can be drawn. It is pivotal to further explore oral manifestations of these conditions bridging the gap between dental care and general medicine to optimise diagnostics, treatment strategies, and ultimately patient care.

## Introduction

Traditionally, gastroenterologists focus on the area from the esophagus to the anus, while the oral cavity has been the domain of the dentist. When systemic conditions of the gastrointestinal tract manifest in the oral cavity also, close collaborations between dental and medical practice are required. The presence of extra-intestinal manifestations of the oral mucosa in the non-infectious chronic diarrhoeal conditions namely, Morbus Crohn (MbC), ulcerative colitis (UC), and coeliac disease (CeD) is well-established, where oral mucosal manifestations may precede intestinal involvement by many years and can be more severe during active intestinal disease [1].

Specific lesions in MbC are, for example, diffuse labial and buccal swelling, cobble stoning, mucosal tags, deep linear ulcerations, mucogingivitis and granulomatous cheilitis [2]. The non-specific lesions are thought to be caused by the adverse effects of MbC, for example, inflammatory reactions caused by MbC, but also malabsorption and nutritional deficiency resulting from chronic diarrhoea, overgrowth of intestinal bacteria, gastrointestinal surgery, or side effects of drug therapy [3]. Non-specific lesions are, for example, aphthous ulcerations, pyostomatitis vegetans, gingivitis, angular cheilitis, glossitis, and gingival hyperplasia [2]. Pyostomatitis vegetans is considered a specific, extra-intestinal manifestation of UC [4, 5]. Non-specific complications include lichenoid lesions, halitosis, altered sense of taste, xerostomia, coated tongue, gingivitis, and periodontitis [4, 5]. The most well-known oral manifestations of CeD are symmetric enamel defects and aphthous lesions, which are more prevalent in children compared to adults, with an incidence of up to 46% [6]. Malabsorption and deficiencies in iron, folic acid, and vitamin B_12_ appear to play a role in the pathogenesis [7]. Other oral mucosal manifestations in CeD are angular cheilitis, oral lichen planus, salivary gland dysfunction, atrophic glossitis, burning sensation of the tongue, and geographic tongue [8].

As irritable bowel syndrome (IBS) and microscopic colitis (MC) are also non-infectious chronic diarrhoeal diseases that affect the gastrointestinal tract and may cause similar systemic effects, it is likely that oral manifestations are also present in these conditions and not only in MbC, UC, and CeD. For example, malabsorption and nutritional deficiencies, as well as bacterial dysbiosis, thought to be the culprits in some of the above-mentioned oral manifestations of IBD and CeD, can be seen in patients with IBS [9–11]. Furthermore, in MC a similar inflammatory reaction can be observed as in MbC and UC, and it has been theorised that MC could in fact be part of the spectrum of autoinflammatory conditions such as IBD involving a Th1 mediated immune reaction [12]. There is also a bidirectional overlap of IBS in CeD and IBD, and patients in remission from CeD or IBD can exhibit IBS-like symptoms [13]. Because of this connection between the non-infectious chronic diarrhoeal diseases, oral manifestation classically attributed to a specific disease might in fact also be connected to the others. Yet, the occurrence of oral manifestations in IBS and MC, is currently poorly explored.

IBS is a functional gastrointestinal disorder of the small and large intestine causing a chronic abdominal pain associated with a change in the frequency or form of stool where the pathophysiology is not yet clearly understood [14]. However, it is likely a disorder of gut-brain interaction involving factors such as visceral hypersensitivity, abnormal gastrointestinal motor function, altered gastrointestinal mucosal and immune function, abnormalities in the gut microenvironment, and altered CNS processing [15, 16]. Risk factors for this condition include female gender, stress, somatisation, and abdominal obesity [17]. Disease onset is suggested to be triggered by stress, gastrointestinal infection, or abdominal surgery, causing alterations of the enteric nervous system, and leading to irregular secretory-, sensory- and motor function of the gastrointestinal tract in genetically predisposed individuals [18]. Diagnosis is currently based on the Rome IV criteria for functional gastrointestinal disorders, revised by Mearin et al. [19]. The prevalence of IBS using the Rome IV criteria is approximately 4.1% worldwide, with females displaying a higher prevalence than men. IBS is also often seen together with chronic pelvic pain, chronic fatigue, fibromyalgia, and food intolerances, as well as other functional gastrointestinal disorders such as functional dyspepsia, gastroesophageal reflux disease, nausea, and incontinence [17]. IBS-like symptoms are also commonly seen in IBD and CeD [20].

MC is a chronic inflammatory disease affecting the colon and is associated with symptoms such as watery diarrhoea, abdominal pain, faecal incontinence, and arthralgia [21]. Depending on the severity of the condition, patients may experience weight loss and fatigue, as well as reduced quality of life and depression [22]. For diagnosis, a colonic biopsy and histological analysis is necessary. Histologically, chronic inflammation of the lamina propria and surface epithelium injury can be seen [23]. Further histological findings distinguish the two subgroups of MC, lymphocytic colitis (LC) and collagenous colitis (CC) [22]. The pathophysiology of MC is not yet clearly understood, but it seems to be multifactorial [23]. Increased permeability of the intestinal barrier in combination with an atypical immune reaction to agents in the colonic mucosa or lumen may account for the development of the condition [22]. As familial cases have been observed, some individuals may have a genetic predisposition [23]. Risk factors include smoking, female gender, older age, drug exposure, and autoimmune disorders [21]. Smoking increases the risk by three to five times and advances disease onset significantly [22]. The incidence of MC increases with age; on average, patients receive the diagnosis in their sixties [24]. There is a strong female predominance, with females being three to four times more likely to develop MC [24]. There is also a strong association between MC and exposure to certain drugs, such as nonsteroidal anti-inflammatory drugs (NSAIDs), proton pump inhibitors (PPIs), and selective serotonin reuptake inhibitors (SSRIs), with recent and prolonged exposure increasing the risk of developing drug-induced MC [22]. Autoimmune conditions such as CeD, rheumatoid arthritis, and type 1 diabetes mellitus are commonly seen in patients with MC, and it has been theorised that autoimmunity may play a role in the development of the condition [23].

The overall objective of this study was to review the current knowledge of possible associations between manifestations and diseases of the oral mucosa and salivary glands in IBS and MC. In addition, we explored the possible mechanisms behind the oral symptoms and aimed to highlight any knowledge gaps related to this topic.

## Materials and methods

### Study design

The study was designed closely following the PRISMA (Preferred Reporting Items for Systematic reviews and Meta-Analyses) guidelines [25]. Also, a protocol using the Population, Intervention, Comparison, Outcome (PICO) framework was used to describe the planned methods of the study improving precision and transparency, and to minimise the risk of bias. The protocol adhered to the PROSPERO guidelines (PROSPERO, CRD42022319904) [26]. This review involved a systematic search of the literature based on medical subject headings (MeSH), keywords, and free-text terms, then screening of literature titles and abstracts using specific inclusion and exclusion criteria according to the PICO framework, and thereafter classifying the internal validity (quality) of the included studies, extraction of data from the selected studies, and synthesis of the evidence.

### Information sources

The electronic bibliometric search was carried out in two different databases; PubMed (maintained by MedLine) and Scopus (maintained by Elsevier). The last search was conducted on 18th February 2025. Other databases (Google Scholar and Cochrane libraries) presented only with duplicates and articles not relevant for the study. When using citation searching, no further articles were retrieved. The overall search strategy was developed together with librarians at the University of Gothenburg’s Biomedical Library to maximise the sensitivity.

### Literature search strategy

Searches were made using MeSH, keywords, and free-text terms in the title/abstract, adapted to fit each database separately. The search strategy was compiled from general and specific search terms based on clinical experience as well as results from test searches. The complete search syntax for PubMed and Scopus is presented in Appendix 1.

### Study selection

**Figure 1:**
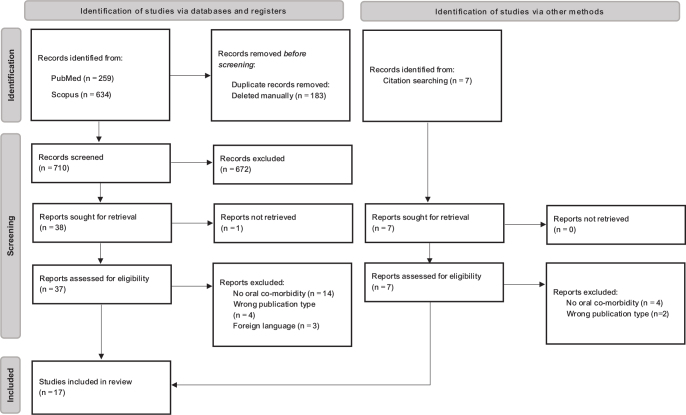
The PRISMA flow diagram for the systematic review shows the different phases of identification and selection of the studies for inclusion.

The online systematic review collaboration tool Rayyan [27] was used to collect and upload all identified articles and their abstracts. Duplicates were detected automatically by the tool and removed manually. Screening was performed independently at title and abstract level by two researchers (HG and MB) against the inclusion criteria defined for the review. Thereafter, the decisions were de-blinded and cross-referenced. Conflicting decisions were discussed until consensus was reached. Where required, the full text was retrieved and read before a decision was reached. The full text of selected studies was retrieved and assessed in detail against the inclusion criteria by the two independent reviewers. Full-text studies that did not meet the inclusion criteria were excluded, and reasons for exclusion were noted. Furthermore, the reference lists of all studies selected for critical appraisal were screened for additional studies to retrieve. Finally, citation mining was performed using the literature mapping tool ResearchRabbit [28]. The bibliometric review included case-control studies, cohort studies, cross-sectional studies, and case reports. The search results are presented in the PRISMA flow diagram, Figure 1. Excluded database articles after full-text review are presented in Appendix 2.

### Eligibility criteria

As no specific intervention (I) or comparison (C) was to be studied, and outcome (O) was hard to define as it could be both oral manifestations/disease or IBS/MC diagnosis, depending on the study design, the focus for assessing eligibility was population (P), according to the PICO framework. All inclusion and exclusion criteria are presented in Table 1. The inclusion criteria entailed patients with a diagnosis of IBS or MC with oral manifestations/disease, of any age, diagnosis, disease severity, ethnicity, gender, geographical area, socio-economic status, and type of treatment for the disease. Only full-length original articles in the English, Swedish, Norwegian, and Danish language were included.

**Table 1:** Inclusion and exclusion criteria for eligibility assessment.

Inclusion criteria	Exclusion criteria
Patients with a diagnosis of IBS or MC with oral manifestations/disease, of any:	
Age	Animal studies
Diagnosis	Article retractions
Disease severity	Foreign language
Ethnicity	Grey literature
Gender	*In vitro* studies
Geographical area	Letters or comments
Socio-economic status	Secondary sources (books, reviews)
Type of treatment for the disease	Unavailable, full‐text studies
Full-length original articles in the English, Swedish, Norwegian, and Danish language only	

Exclusion criteria included animal studies, article retractions, foreign language, grey literature, *in-vitro* studies, letters or comments, secondary sources (books, reviews), and unavailable, full-text studies.

### Quality assessment

This was performed using the Joanna Briggs Institute’s Critical Appraisal Tool for Systematic Reviews [29], consisting of different checklists for each study type. Two researchers (HG and MB) independently assessed all included articles. As the articles each had a different focus, exposure was defined as the pre-existing condition described in the inclusion criteria for the study and outcome of the condition of interest. The decisions were then cross-checked, and conflicting assessments were discussed and reviewed. Appraisal scores were calculated as the number of quality points received out of possible points for each respective study type, expressed as a percentage. Quality decisions and appraisal scores are presented in Appendices 3–6 for Analytical Cross-Sectional Studies, Case-Control Studies, Cohort Studies, and Case Reports, respectively; and presented in the results section. Due to the limited material available for this topic, all studies were included regardless of quality.

### Data extraction

A data extraction table modified from the Joanna Briggs Institute SUMARI Data Extraction Tool [30], was used. The extracted characteristics were modified to fit all types of included studies and clarified to enable a better overview. Data extraction was performed by one researcher (HG) and checked by another researcher (MB). The complete data extraction tables are presented in Tables 2 and 3. Only data relevant to the study were extracted. To be able to compare the studies more clearly, the data were reported in the same way across all studies, where possible. However, there are exceptions as not all studies presented their data in the same way.

**Table 2:** Data extraction – Characteristics of included studies of IBS.

Study	Country	Study design	Setting/context	Sample size (subjects/controls)	Population (sex/age)	Diagnosis/diagnostic criteria	Methodology	Variables/outcomes of interest	Description of main results
Andréasson et al. (2016)	Sweden	Cross-sectional	University hospital, rheumatology clinic	***Patient group:*** 56 patients ***Control group:*** 29 controls	***Patient group:*** 2 males, 54 females Mean age 62 years (range 53–68) ***Control group:*** 3 males, 26 females Mean age 56 years (range 49–58 years)	***Patient group:*** Diagnosis of primary SS based on the American-European consensus group criteria ***Control group:*** Recruited from hospital staff without rheumatological or gastrointestinal disease	Health questionnaire (Rome III and VAS-IBS) Medical record review	Diagnosis of IBS and severity	16 patients (29%) with SS suffered from IBS. Patients with SS and IBS experienced more pain than those without IBS.
Barton et al. (1999)	United Kingdom	Case- control	University hospital, gastroenterology department	***Patient group:*** 46 patients ***Control group:*** 46 controls	***Patient group:*** 8 males, 38 females Mean age 44 years (range 20–70 years) ***Control group:*** Age- and sex- matched controls	***Patient group:*** IBS diagnosis based on the Rome criteria ***Control group:*** Recruited from hospital staff, with no history of gastrointestinal complaints	Health questionnaire Schirmer test for tear production Rose-Bengal testing for sicca Detection of antibodies towards extractable nuclear antigen (Ro and La)	Diagnosis of SS (based on subjective and objective symptoms)	Dry mouth was more frequent in patients with IBS (25/46; 54%) than in the control subjects (4/46; 9%) (*p* < 0.001). Patients with IBS had a significantly higher prevalence of SS than control subjects (6% vs. 33%, *p* = 0.004).
Canataroǧlu et al. (2001)	Turkey	Case- control	University hospital, gastroenterology department	***Patient group:*** 78 patients ***Control group:*** 70 controls	***Patient group:*** 24 males, 54 females Mean age 40.9 ± 11.1 years ***Control group:*** 24 males, 46 females Mean age 39.4 ± 11.7 years	***Patient group:*** IBS diagnosis based on the criteria of Manning ***Control group:*** Undisclosed	Health questionnaire	Self-reported SC (dry mouth/dry eyes)	SC was present in 15% of patients with IBS and in 0% of the subjects in the control group (*p* < 0.001).
Cheung & Trudgill (2015)	United Kingdom	Case report	General hospital, gastroenterology department	1 patient	64-year old female	Past medical history included IBS	Patient interview Medical record review	Presenting with intense burning pain affecting her tongue tip, mouth and lips for the past 5 years	BMS diagnosis (idiopathic).
Erbasan et al. (2017)	Turkey	Cross-sectional	Training and research hospital, department of gastroenterology	77 patients	11 males, 66 females	IBS diagnosis based on the Rome III criteria, excluding organic disease	Patient interview Minor salivary gland biopsy Ophthalmic evaluation (Schirmer test, tear film, tear break-up time, corneal and conjunctival epithelial staining) Antinuclear antibodies	Self-reported dry mouth and dry eyes Focus score ≥1 (positive salivary gland biopsy) Objective dry eyes Positive antinuclear antibodies indicating autoimmune disease	29 patients (38%) with IBS suffered from dry mouth. From this group, 2 patients (3%) presented with a positive salivary gland biopsy and were ultimately diagnosed with SS.
Kim-Lee et al. (2015)	U.S.	Cross-sectional	University hospital, adult autoimmune disease clinic	***IBS group:*** 9 patients ***CeD group:*** 1 patient	2 males, 8 females Mean age 48.2 years (range 28–63 years)	Diagnosis of SS based on the American European Consensus Criteria Diagnosis of IBS	Food specific IgG Elimination diet Provocation diet	Food hyper-sensitivities	All patients with IBS presented with food hypersensitivities (most commonly to wheat and dairy products). Symptoms of IBS improved after eliminating culprit foods, re-institution of culprit foods triggered recurrence of all symptoms.
Lidén et al. (2008)	Sweden	Case- control	University hospital, outpatient rheumatology clinic	***Patient group:*** 21 patients ***Control group:*** 18 controls	***Patient group:*** 2 males, 19 females Mean age 56.0 years (range 34–73 years) ***Control group:*** 13 males, 5 females Mean age 34.0 (range 19–58)	***Patient group:*** Primary SS diagnosis based on the revised American European Consensus Criteria ***Control group:*** Healthy controls	Health questionnaire	Diagnosis of IBS based on the Rome III criteria	13 patients (62%) with primary SS were diagnosed with IBS. 4 of them had diarrhoea-predominant IBS, 2 had constipation-predominant IBS, 1 had alternating IBS and 1 had unsubtyped IBS.
Maxton et al. (1991)	United Kingdom	Cross-sectional	University hospital, outpatient clinic, gastroenterology department	***IBS group:*** 107 patients ***Other GI-diseases:*** 50 UC patients 62 MbC patients 60 peptic ulceration patients 81 gall stone patients 42 reflux patients	***IBS group:*** 22 males, 85 females Mean age 43.6 ± 12.3 for males, 39.4 ± 12.1 for females ***Other GI-diseases:*** 146 males, 149 females in total Mean age 45.5 ± 16.4 for males, 49.1 ± 15.4 for females	***IBS group:*** Diagnosis of IBS based on the occurrence of abdominal pain and distension, abnormal bowel habits and normal laboratory tests ***Other GI-diseases:*** Diagnosis of either UC, MbC, peptic ulceration, gall stones or reflux oesophagitis	Health interview	Self-reported bad breath	65% of IBS patients had subjective bad breath.
Whorwell et al. (1986)	United Kingdom	Case- control	University hospital, outpatient clinic department of medicine	***Patient group:*** 100 patients ***Control group:*** 100 controls	***Patient group:*** 10 males, 90 females Age range 18–64 years ***Control group:*** Age- and sex- matched controls	***Patient group:*** Diagnosis of IBS on the basis of irregular bowel habits, distension and pain with no evidence of organic disease ***Control group:*** Recruited from local industry and commerce staff, screened to exclude IBS	Health interview	Self-reported oral symptoms (sore mouth, mouth ulceration, bad breath/unpleasant taste in mouth)	No significant difference in frequency of sore mouth or mouth ulcerations in patients with IBS compared to control subjects. Bad breath/unpleasant taste in mouth was significantly higher (*p* < 0.0001) in IBS patients (58%) compared to controls (10%).
Zimmerman (2003)	Israel	Case- control	University Hospital, tertiary care gastroenterology department	***IBS group:*** 53 patients with IBS ***IBD group:*** 55 patients with IBD ***Control group:*** 56 controls	***IBS group:*** 53 males Mean age 33.9 ± 1.9 years ***IBD group:*** 55 males Mean age 33.4 ± 1.7 ***Control group:*** 56 random age- and sex- matched controls Mean age 37.5 ± 2.0 years	***Patient group:*** Diagnosis of IBS based on the Rome I criteria attending the gastroenterology clinic Diagnosis of IBD based on clinical criteria ***Control group:*** Hospital students and administrative staff	Health questionnaire	Self-reported oral symptoms (bad breath, foul taste, dryness)	Patients with IBS showed a significantly higher frequency of oral symptoms than both patients with IBD and control subjects (*P* < 0.001).

BMS: burning mouth syndrome; CeD: coeliac disease; IBD: inflammatory bowel disease; IBS: irritable bowel syndrome; SC: sicca complex; SS: Sjögren’s syndrome/sicca syndrome.

**Table 3:** Data extraction – Characteristics of included studies of MC.

Study	Country	Study design	Setting/context	Sample size (subjects/controls)	Population (age/sex)	Diagnosis/diagnostic criteria	Methodology	Variables/outcomes of interest	Description of main results
Andréasson et al. (2016)	Sweden	Cross-sectional	University hospital, rheumatology clinic	***Patient group:*** 56 patients ***Control group:*** 29 controls	***Patient group:*** 2 males, 54 females Mean age 62 years (range 53–68) ***Control group:*** 3 males, 26 females Mean age 56 years (range 49–58 years)	***Patient group:*** Diagnosis of primary SS based on the American-European consensus group criteria ***Control group:*** Recruited from hospital staff without rheumatological or gastrointestinal disease	Medical record review	Diagnosis of MC	1 patient out of 56 (2%) with SS suffered from MC.
Barco et al. (2010)	Spain	Case report	General hospital, dermatology department	1 patient	75-year old female	Past medical history included a diagnosis of CC	Patient interview Medical record review	Patient presented with oral ulcers	Oral ulcers disappeared within a week after treatment with oral prednisone.
Barta et al. (2005)	Hungary	Cross-sectional	University hospital, department of pathology	53 patients	***CC group:*** 21 males, 25 females Mean age 51.6 years ***LC group:*** 2 males, 5 females Mean age 43.4 years	46 patients with CC and 7 patients with LC based on histological examination	Medical record review	Comorbidity with SS	5 patients (9%) had SS, of which all had CC of the constipation subtype.
Melchor et al. (2020)	Spain	Cross-sectional	Multicentre, rheumatology centres across Spain	437 patients	22 males, 415 females Median age 58 years	Patients from the Sjögrenser study with a diagnosis of primary SS based on the European-American consensus criteria	Patient interview Medical record review	Digestive involvement Diagnosis of LC	3 patients (1%) with SS suffered from LC
Mohammed et al. (2022)	U.S.	Cohort	Medical center, department of gastroenterology and hepatology	***Patient group:*** 1130 patients ***Control group:*** Undisclosed	***Patient group:*** 250 males, 880 females Aged 18 or older ***Control group:*** Undisclosed	***Patient group:*** Patients with diagnosis of MC ***Control group:*** Patients without diagnosis of MC	Medical record database review	Comorbidity with SS	20 patients (2%) with MC had SS, the odds ratio was 10.8. SS is a risk factor for MC.
Soulier et al. (1996)	France	Cross-sectional	University hospital, rheumatology department	***Patient group:*** 7 patients ***Control group:*** 7 controls	***Patient group:*** 2 males, 5 females Mean age 56 years (range 33-76 years) ***Control group:*** Age-matched controls Mean age 55.4 years	***Patient group:*** Diagnosis of CC based on histological examination ***Control group:*** Controls without SS	Patient interview Minor salivary gland biopsy Immunologic testing	Simplified criteria of Vitali (subjective sicca symptoms, histopathological findings, autoantibodies)	4 patients were diagnosed with SC as they reported dryness of the eyes (*n* = 2) and/or mouth (*n* = 3). However, none met the criteria by Vitali and thus could not be diagnosed with SS.
Vigren et al. (2013)	Sweden	Cross-sectional	Multicentre, outpatient clinics at university hospitals	116 patients	24 males, 92 females Median age 62 years (range 55–73 years)	Patients with a diagnosis of CC	Health questionnaire Medical record review	Comorbidity with SS	4 patients (3%) with CC suffered from SS.
Widgren & MacGee (1990)	Switzerland	Case report	University hospital, department of pathology	1 patient	81-year-old female	Past medical history included SS	Patient interview Medical record review	Presenting with persistent diarrhoea and abdominal cramping with varying intensity over 13 years	The patient was diagnosed with CC based on histological examination.

CC: collagenous colitis; LC: lymphocytic colitis; MC: microscopic colitis; SC: sicca complex; SS: Sjögren’s syndrome/sicca syndrome.

## Results

### Description of included studies

In total, 893 articles were retrieved from the two databases PubMed and Scopus. After removing duplicates, 710 articles remained. Only 38 articles passed the abstract screening, of which 16 fulfilled the inclusion criteria and were eligible for inclusion after full-text screening. The reasons for exclusion of the other articles are presented in the PRISMA diagram, Figure 1 and Appendix 2. Seven additional articles of interest were identified through citation searching. Ultimately, only one additional article was included, as none of the others fulfilled the inclusion criteria. Out of the 17 included studies, eight were cross-sectional studies (47%), five were case-control studies (29%), one was a cohort study (6%), and three were case-reports (18%), of various methodologies. All of them were retrospective.

The methodological quality deviated greatly, ranging from 13 to 100% between and within study types. Generally, the articles did not identify and/or did not have strategies to deal with confounding factors. Case reports were mostly of poor quality, failing to describe the patients in sufficient detail. Because of the low number of included studies, no articles were excluded after quality assessment regardless of appraisal score.

The included studies were conducted between 1986 and 2025. Three studies were published from Sweden and the United Kingdom, respectively. The remaining papers were from a range of different countries. All but three articles (one from Israel and two from the United States of America) were from European countries. A total of 10 articles studied IBS and eight studied MC, since one article depicted both the conditions. All studies included patients from a hospital environment, whereof seven studies were conducted in gastroenterology departments and five were in rheumatology departments specifically.

Most of the data were retrieved from patient interviews, health questionnaires, and medical records. Sample sizes ranged from 1 to 107 for IBS and 1–1,130 individuals for MC, where nine studies provided a control group. In total, the study population consisted of 2,350 patients, of which 500 (21%) were diagnosed with IBS and 1,312 (56%) with MC. The remaining patients had some type of oral manifestations but no diagnosis of IBS or MC, or were diagnosed with another gastrointestinal disorder. There was a dominance of females included in the study populations, in total 1,894 (81%) females for all studies compared to 457 (19%) males. For studies on IBS, 76% were females and for MC 82% were females. The age ranged from 18 to 73 years in studies of IBS and from 18 to 81 years in studies of MC. The articles included studies reporting oral disease in IBS or MC, or both. Consequently, study populations included either patients with a diagnosis of IBS or MC, or patients with oral disease. Therefore, the measured outcome of comorbidity could either be in the form of oral symptoms (diagnosis of Sjögren’s syndrome [SS] or self-reported dryness of mouth and eyes, or self-reported oral symptoms) or diagnosis of IBS or MC. The complete data extraction with study characteristics is presented in Tables 2 and 3.

### Irritable bowel syndrome

Two articles explored the prevalence of IBS in patients with SS; 29% [31] and 62% [32], respectively. Another article investigated if patients with SS experienced gastrointestinal symptoms because of food hypersensitivities, and found that an elimination diet improved symptoms in all nine patients diagnosed with IBS [33].

Conversely, three articles explored the prevalence of SS and/or sicca complex (SC) in patients with IBS. In the first article, a total of 54% of patients with IBS suffered from dry mouth, which was significantly higher than the prevalence of 9% in the controls (*p* < 0.001) and 33% of the patients with IBS were diagnosed with SS compared to 6% of the controls (*p* = 0.004) [34]. In the second article, 15% of patients with IBS suffered from SC compared to the controls, where none suffered from SC [35]. Finally, in the third article, dry mouth was identified in 38% of the patients, of which two patients (3%) were later diagnosed with SS [36].

Oral manifestations besides SS and SC were reported in a few of the articles, such as a case report where a woman diagnosed with IBS was later also diagnosed with burning mouth syndrome (BMS) [37]. Sore mouth and mouth ulcerations were reported in one article. However, there was no significant difference between the patients with IBS and the controls [38]. When looking at bad breath, foul taste and dryness combined, the occurrence was significantly higher in patients with IBS compared to both patients with IBD and controls (*p* < 0.001) [39]. Bad breath was reported in two more articles. The prevalence was significantly higher (*p* < 0.0001) for patients with IBS than controls (58% compared to 10%) [38]. This was also true for patients with IBS compared to other gastrointestinal diseases (UC, MbC, peptic ulceration, and gall stones), with a reported prevalence of 65% in IBS and 45% overall for the other diseases (*p* < 0.01) [40].

### Microscopic colitis

Two articles explored the prevalence of MC in patients with SS, where one out of 56 patients (2%) [31] and three out of 437 patients (1%) [41] with SS also suffered from MC. One case report presented a woman with SS who was later diagnosed with CC [42].

Conversely, three articles explored the prevalence of SS in patients with MC presenting 2% [43], 3% [44], and 9% [45], respectively. One article reported sicca symptoms in 57% (4 out of 7) of patients with CC, whereof three patients experienced dry mouth and two dry eyes; yet, none of the patients met the criteria of SS [46].

Regarding other oral symptoms, one case report described a woman diagnosed with CC presenting with oral ulcers [47].

## Discussion

Currently, non-infectious chronic diarrhoeal conditions present an enormous burden on healthcare systems and national economies worldwide. Since oral mucosal manifestations may precede intestinal involvement by many years, recognising features of the conditions in the oral cavity, may help to establish an earlier and more accurate diagnosis of the underlying disease. It can also enable more targeted treatment of the oral symptoms that may otherwise go undetected, thus contributing to a better quality of life for patients. Also, identifying oral manifestations can help deepen our understanding of the pathophysiological mechanisms. Since oral manifestation of IBS and MC have not been as vastly explored as the other non-infectious chronic diarrhoeal conditions namely IBD and CeD, this was the aim of the current study.

The prevalence of IBS in patients with SS ranged from almost 1/3rd to 2/3rd, where eliminating culprit foods may alleviate symptoms. Conversely, the prevalence of SS in patients with IBS presented in up to 1/3rd of patients. Sicca complex presented in 15% of patients with IBS and dry mouth in up to 50% of patients. Furthermore, BMS may present even if the evidence currently is sparse. No significant difference in the occurrence of oral ulcers in patients with IBS versus controls was found. The occurrence of bad breath and foul taste was reportedly significantly higher in patients with IBS where the prevalence for bad breath was approximately 60%. The prevalence of MC in patients with SS was 1% to 2%, and the presence of SS in patients with MC ranged from 2% to 9% which are both lower than the results presented for IBS and SS. Dry mouth in patients with MC reached approximately 40% which is similar to the results presented for IBS. The presence of oral ulcers is difficult to evaluate since this was only presented in one case report. There is no other evidence in the literature for other oral manifestations or symptoms in MC.

Appraisal scores varied considerably between studies. For the articles on IBS, the scores ranged from 13% to 70%, with a mean score of 46%. Conversely for MC, the appraisal score ranged from 13% to 100%, with a mean score of 54%. The articles generally did not identify or deal with confounding factors which may influence the results. As an example, not all the articles that explored dry mouth considered the medications or conditions of the included patients which may contribute to this symptom. Furthermore, for the cross-sectional studies, the more poorly ranked articles did not describe the sample inclusion criteria and setting sufficiently. Similarly, the case-control studies with the lowest appraisal scores did not appropriately match and describe the groups. Failing to do so, limits the possibility to evaluate the study and/or increases the risk of selection bias. As we do not know how the groups were selected and from what population, generalising the results might not be possible. As such, the results of the articles with poorer quality should be critically reviewed. Since included articles were published over a long period of time, from 1986 to 2025, the criteria used for the diagnosis of IBS varied. Also, some of the articles did not specify the diagnostic criteria. This could lead to misclassification bias between studies, as one patient could have IBS based on one criterion but not another, making the results less reliable. As this area of medicine is relatively unexplored, case reports were included to help identify possible oral manifestations of IBS and MC respectively. Furthermore, considerations must be made regarding the aims of the included studies. In many cases, oral manifestations were not the focus. Generally, the oral manifestations were not brought up in the discussion, and no conclusions were made on the possible causality.

### Sjögren’s syndrome, sicca syndrome, dry mouth, and halitosis

Based on the results of this study, an association between IBS and SS with a potential bidirectional relationship could be possible. However, both IBS and SS symptoms can be discreet and may be overlooked, implying that one disease may precede the other but with diagnosis being delayed until after onset of the other disease. It is unclear if the relationship is causal or if the diseases share a common pathophysiology. Interestingly, in the study by Kim-Lee et al. [33], all patients with IBS and SS had some type of food hypersensitivity, which seemed to account for their symptoms of IBS. This raises an interesting question as to whether these symptoms in some patients might be due to undiagnosed hypersensitivities, and if ruling this out should be a step for diagnostics of IBS in the future. While this study had a limited sample size, the occurrence of food hypersensitivity was higher than for patients with IBS in general, where between 20 and 65% had food hypersensitivities [48]. Overall, patients with SS have an increased risk of developing gastrointestinal symptoms [49]. As such, further exploring this possible connection could better help our understanding of the diseases and improve management of these patients. The results also suggest a connection between MC and SS. Interestingly, comorbidity of IBD and SS has not been described as often in the literature as was found in the current study for IBS and MC. Most published articles on IBD and SS are case reports [50], and an association has been deemed unlikely [51].

The results of the current study indicate that dry mouth is common in IBS; however, a causal relationship seems unconvincing at the moment. None of the articles specified how the patients were asked about dry mouth symptoms, how often and when the symptoms occurred. No articles were identified that measured salivary flow rates and investigated possible hyposalivation. He et al. [52] found that the secretion of salivary alpha-amylase is abnormal in patients with IBS, with a higher concentration in resting saliva and a lower concentration in stimulated saliva than in healthy controls. This suggests altered sympathetic activity in patients with IBS that could also affect the production and secretion of other salivary components such as mucins, thus altering saliva composition. If this is the case, it could be explanatory as to why patients with IBS experience dry mouth. Also, this symptom is commonly seen in patients with anxiety and depression [53], which in turn are common conditions seen in patients with IBS [54]. Both Whorwell et al. [38] and Maxton et al. [40] reported a high prevalence of subjective bad breath (halitosis) in patients with IBS. Bad breath and foul taste can be quite unspecific symptoms, as they are subjective and therefore cannot be measured. Also, smell and taste perception can be altered by many factors and may have many different causes. However, this association seems probable, as several symptoms associated with IBS are known to cause bad breath in certain individuals, and gastrointestinal pathology has been identified as a common culprit in halitosis [55]. Gastroesophageal reflux disease, commonly seen in patients with IBS [56], is a clear risk factor for subjective bad breath [57]. Therefore, asking patients with halitosis about gastrointestinal symptoms could be an indicator for gastrointestinal diseases such as IBS in the future. This also highlights the need for dentists to have greater medical knowledge of systemic diseases and be observant of patients’ subjective complaints, as well as the importance of coordination between dental care and general practice.

### Oral ulcers

For IBD and CeD, there is a strong correlation even though the pathophysiology is unclear [58]. This correlation is virtually unexplored for IBS and MC, therefore more research is advocated. Interestingly, Kiliç et al. [59] reported that patients with recurrent aphthous stomatitis (RAS) have a high prevalence of various gastrointestinal symptoms, such as stomach aches and diarrhoea, which could possibly originate from unidentified gastrointestinal disease. Also, IBS and MC are relatively recently established diseases, with the first case of CC being described in 1976 [60], and the first criteria for IBS diagnosis being developed in 1978 [61]. This may account for why presently, so few cases and/or studies have described oral ulcers or oral manifestations in general in these conditions. Based on our results, we cannot draw a conclusion regarding a possible association between IBS or MC, and oral ulcers.

### Burning mouth syndrome

Burning mouth syndrome was only described in one case report in a patient with IBS, where it was deemed idiopathic, and we therefore have no evidence for this association from the current systematic review. However, a possible association cannot be excluded. For IBD and CeD, there are only a few articles reporting comorbidity with BMS [62–64]. Possible systemic factors in BMS include nutritional deficiency of vitamin B, iron, and zinc [65], which are also thought to contribute to the development of aphthous stomatitis commonly seen in IBD and CeD [66, 67]. This apparent connection should warrant further investigation of the occurrence of BMS in gastrointestinal diseases, which is currently vastly unexplored.

### Possible pathophysiological mechanisms

The increased prevalence of xerostomia found in our study could be attributed to somatisation, manifesting feelings leading to physical reactions as well as medications. This is commonly seen in individuals in general, for example, sweating or elevated heart frequency because of nervousness. However, it seems more pronounced in patients with IBS and is thought to be one of the mechanisms behind the disease [68]. This phenomenon occurs when the sympathetic nervous system shuts down functions deemed unnecessary in stressful situations. For healthy individuals, this may only happen in highly stressful circumstances. However, patients with IBS may have a lower threshold, resulting in dry mouth being experienced in less stressful situations and more often. Another feature in patients with IBS is over-attention to body symptoms [68]. Heightened sensitivity and excessive disease attribution may lead to these patients being bothered by and reporting dry mouth more often than healthy individuals. The study by Andreasson et al. [31] found that patients with SS and IBS experienced more pain than those without IBS, which may point to this heightened sensitivity. This could be explained by the current theory that these patients experience insufficient inhibition of incoming pain signals, meaning that unnecessary, low-grade input from nerves throughout the body continuously reaches the brain and is registered as pain [68].

An association between MC and other autoimmune disorders has previously been described, suggesting that there might be an autoimmune aspect of this condition [69]. It is well established that autoimmune disorders often show comorbidity and may aggregate in one individual or family [70]. For example, CeD and SS can be seen together frequently, seemingly due to their common autoimmune background [71]. This could be a common feature also in MC and SS, accounting for the comorbidity seen in our study.

There are studies that suggest that the oral microbiome might influence the development of gastrointestinal disease. A clear relationship between the oral and gastrointestinal microbiome has been reported, where some oral microbes disrupt and outcompete the normal duodenal flora giving rise to gastrointestinal symptoms [72]. Furthermore, oral microbes in patients with IBS may be a promising factor for diagnostics and further understanding of the disease [73]. Also, the oral species *Campylobacter concisus* has been proposed as a possible cause of gut inflammation in patients with MC [74]. Identifying oral bacteria with the potential to cause intestinal dysbiosis or commonly seen in IBS and MC would be an interesting addition.

Originally, this study aimed to compile the current knowledge of oral manifestations in all the non-infectious chronic diarrhoeal conditions, including IBD (MbC and UC) and CeD as well as IBS and MC. Despite the already established connection between certain oral manifestations in IBD and CeD, other possible manifestations may be less explored. Also, there were no systematic reviews exploring all oral manifestations in CeD, only specific lesions such as aphthous stomatitis. However, given the overwhelming number of articles resulting from this search, it was impossible to compile all these results within the scope of this study. Also, a similar systematic review compiling oral manifestations in IBD was done in 2019 [75]. Thus, the aim was limited to including IBS and MC since oral manifestations in these conditions have been poorly studied.

Performing a meta-analysis might have given the study greater validity as well as enabling us to draw more accurate conclusions regarding the results. However, since the included studies showed great heterogeneity, meta-analysis would have to be performed separately for each association and would not be possible for associations that were only described in one included study or case report. As not all the articles had control groups or reported the frequency of oral manifestations/disease in these groups, pooled prevalence seems to be the most fitting possible analysis. Also, the aims and study designs varied greatly. Performing meta-analysis on a limited set of studies can sometimes lead to an illusion of certainty and may be misleading [76]. Thus, no considerations were made about other possible causal factors that might be presented in the respective study groups [77]. A pooled prevalence would further hide these variables and likely reduce the reader’s critical analysis of the results. For these reasons, the present study reported data separately and made it clear that no definite conclusions about the causality of the associations can be drawn, while still answering the aim of possible oral manifestations and highlighting the need for further research in this area.

Retrospectively, adding more specific search terms to the search syntax would have ensured that no possible oral manifestations were missed. As the theory stated that oral manifestations could be possible in IBS and MC because of their connection to the other chronic non-infectious diarrhoeal disorders prevalently showing oral symptoms, it would be natural to add the specific lesions associated with these conditions in the search syntax. For example, granulomatous cheilitis and pyostomatitis vegetans. Similarly, non-specific lesions, for example, gingival hyperplasia, lingua geographica, and angular cheilitis could have been added as not all the articles used general terms such as ‘oral’ or ‘extraintestinal’. While adding specific search terms might not have generated any additional articles, it would have made our systematic review more thorough and valid.

In the future, it would be beneficial to design studies of a prospective nature to explore possible oral manifestations in IBS and MC. Studying patients with a diagnosis of each of these conditions from a patient history aspect and clinically would give this knowledge. It would also allow a comparison to IBD and CeD, and the oral lesions that they present with. Conducting multi-centre studies comparing parts of the world where the prevalence of these conditions is higher to other parts would help gain a broader understanding. If oral lesions are a part of the array of symptoms in IBS and MC, conducting studies exploring etiopathogenesis is of importance. Ultimately this would be beneficial for patient-centred care and treatment implications guiding healthcare professionals in clinical practice, and allowing patients to better understand their disease and receiving optimal treatment options.

## Conclusions

This systematic review suggests an association between IBS and SS. In addition, a possible correlation between MC and SS has also been identified. The focus of these articles were not oral manifestations in most cases, but extraintestinal manifestations of the disease in general. Only a few articles explored other oral manifestations in IBS and MC. Consequently, no specific conclusions on oral manifestations in these two non-infectious chronic diarrhoeal conditions can be drawn. Overall, the results presented must thereby be interpreted with caution since the quality of the studies were poor and the number of studies too few to draw any definite conclusions. It is pivotal to further explore oral manifestations of these non-infectious chronic diarrhoeal conditions to understand if there are any oral manifestations in IBS and MC, and if there are which oral lesions they present with. There is a well-established association in the other non-infectious chronic diarrhoeal conditions MbC, UC, and CeD, therefore this may also be likely for IBS and MC. This will further bridge the gap between dental care and general practice to optimise diagnostics, patient care, and treatment strategies.

## Supplementary Material


