# Supplementary material for: Manifestations of the oral mucosa and salivary glands in irritable bowel syndrome and microscopic colitis – A systematic review
Source: Acta Odontol Scand. 2025 Jun 11;84:43870. doi: 10.2340/aos.v84.43870 (PMC12186438; doi:10.2340/aos.v84.43870)
Supplement: Supplementary file 1 [file AOS-84-43870-s1.pdf]

Supplementary material has been published as submitted. It has not been copyedited or typeset by Acta Odontologica Scandinavica.

Appendix 1. Search syntax for PubMed and Scopus with the last search conducted 18th February 2025.

| Database | Search Syntax | Results |
|---|---|---|
| *PubMed* | ("colitis microscopic"[Title/Abstract] OR "Microscopic Colitis"[Title/Abstract] OR "collagenous colitis"[Title/Abstract] OR "colitis collagenous"[Title/Abstract] OR "lymphocytic colitis"[Title/Abstract] OR "colitis lymphocytic"[Title/Abstract] OR "colitis, microscopic"[MeSH Terms] OR "colitis, collagenous"[MeSH Terms] OR "colitis, lymphocytic"[MeSH Terms] OR "Irritable bowel syndrome"[MeSH Terms] OR "IBS"[Title/Abstract] OR "Spastic colon"[Title/Abstract] OR "mucous colitis"[Title/Abstract] OR "Spastic bowel"[Title/Abstract] OR "Irritable bowel syndrome"[Title/Abstract] OR "Irritable Bowel Syndromes"[Title/Abstract] OR "syndrome irritable bowel"[Title/Abstract] OR "syndromes irritable bowel"[Title/Abstract] OR "colon irritable"[Title/Abstract] OR "Irritable Colon"[Title/Abstract] OR "colitis mucous"[Title/Abstract] OR "colitides mucous"[Title/Abstract] OR "Mucous Colitides"[Title/Abstract] OR "mucous colitis"[Title/Abstract]) AND ("stomatitis, aphthous"[MeSH Terms] OR "RAS"[Title/Abstract] OR "Recurrent aphthous stomatitis"[Title/Abstract] OR "Aphthous lesion"[Title/Abstract] OR "Aphthous lesions"[Title/Abstract] OR "Recurrent aphthous ulceration"[Title/Abstract] OR "Aphthous Stomatitides"[Title/Abstract] OR "Aphthous Stomatitis"[Title/Abstract] OR "ulcer aphthous"[Title/Abstract] OR "Aphthous Ulcer"[Title/Abstract] OR "Aphthous Ulcers"[Title/Abstract] OR "ulcers aphthous"[Title/Abstract] OR "Canker Sore"[Title/Abstract] OR "Canker Sores"[Title/Abstract] OR "sores canker"[Title/Abstract] OR "Periadenitis Mucosa Necrotica Recurrens"[Title/Abstract] OR "RAS"[Title/Abstract] OR "Recurrent aphthous stomatitis"[Title/Abstract] OR "Aphthous lesion"[Title/Abstract] OR "Aphthous lesions"[Title/Abstract] OR "Recurrent aphthous ulceration"[Title/Abstract] OR "oral manifestations"[All Fields] OR "mouth"[Title/Abstract] OR "oral mucosa"[Title/Abstract] OR "oral cavity"[Title/Abstract] OR "extraintestinal manifestations"[Title/Abstract] OR "extraintestinal manifestation"[Title/Abstract] OR "oral symptom"[Title/Abstract] OR "oral symptoms"[Title/Abstract] OR "oral ulcer"[Title/Abstract] OR "oral ulcers"[Title/Abstract] OR "dry mouth"[Title/Abstract] OR "xerostomia"[Title/Abstract] OR "sicca syndrome"[Title/Abstract] OR "sicca complex"[Title/Abstract] OR "sjogrens syndrome"[Title/Abstract] OR "halitosis"[Title/Abstract] OR "bad breath"[Title/Abstract] OR "saliva"[Title/Abstract] OR "salivary gland"[Title/Abstract] OR "burning mouth syndrome"[Title/Abstract] OR "BMS"[Title/Abstract] OR "xerostomia"[MeSH Terms] OR "Burning Mouth Syndrome"[MeSH Terms]) | 259 |
| *Scopus* | TITLE-ABS ( "collagenous colitis" OR "lymphocytic colitis" OR "microscopic colitis" OR "irritable bowel syndromes" OR "irritable bowel syndrome" OR "irritable | 634 |

| | |
|---|---|
| colon" OR "mucous colitides" OR "mucous colitis" OR ibs OR "spastic colon" OR "nervous colon" OR "spastic bowel" ) AND TITLE-ABS ( "Aphthous Stomatitides" OR "Aphthous Stomatitis" OR "Aphthous Ulcer" OR "Aphthous Ulcers" OR aphthae OR "Canker Sore" OR "Canker Sores" OR "Periadenitis Mucosa Necrotica Recurrens" OR "Recurrent aphthous stomatitis" OR "Aphthous lesion" OR "Aphthous lesions" OR "Recurring oral aphthae" OR "Recurrent aphthous ulceration" OR "oral manifestations" OR "oral symptoms" OR "oral symptom" OR "mouth" OR "oral mucosa" OR "oral cavity" OR "extraintestinal manifestations" OR "extraintestinal manifestation" OR "oral ulcer" OR "oral ulcers" OR "dry mouth" OR "xerostomia" OR "sicca syndrome" OR "sicca complex" OR "sjogrens syndrome" OR "halitosis" OR "bad breath" OR "saliva" OR "salivary gland" OR "burning mouth syndrome" OR "BMS" ) | |

Appendix 2. Excluded articles after full-text review and reason for exclusion.

| **Excluded articles** | **Reason for exclusion** |
|---|---|
| Bermejo et al. (1999) | No-co morbidity |
| Chang & Heitkemper (2004) | Wrong publication type |
| David et al. (2014) | No co-morbidity |
| Fajardo & Pardil (2005) | No co-morbidity |
| Fourie et al. (2016) | No co-morbidity |
| Hedström et al. (2022) | No co-morbidity |
| Jean et al. (1999) | Foreign language |
| Kabir et al. (2009) | No co-morbidity |
| Kilic et al. (2017) | No co-morbidity |
| Krebs (2011) | No-co morbidity |
| Kuzminski et al. (2020) | No co-morbidity |
| Lario et al. (1996) | Foreign language |
| Lembo (2007) | No co-morbidity |
| Levander (2003) | No co-morbidity |
| Malago (2012) | Wrong publication type |
| Ohlsson et al. (2009) | Wrong publication type |
| Prabhu (2014) | Wrong publication type |
| Svistunov et al. (2018) | Foreign language |
| Wiener (1986) | No co-morbidity |
| Yu & Rodriguez (2017) | No co-morbidity |
| Zeiter & Hyams (1997) | No co-morbidity |

Appendix 3. Quality assessment of Analytical Cross-Sectional Studies.

| Citation | Q1 | Q2 | Q3 | Q4 | Q5 | Q6 | Q7 | Q8 | Appraisal score (%) |
|---|---|---|---|---|---|---|---|---|---|
| *Andréasson et al. (2016)* | Y | N | Y | Y | N | N | Y | Y | 63 |
| *Barta et al. (2005)* | Y | Y | Y | Y | N | N | Y | Y | 75 |
| *Erbasan et al. (2017)* | Y | Y | Y | Y | Y | U | N | U | 63 |
| *Kim-Lee et al. (2015)* | N | N | Y | U | N | N | N | U | 13 |
| *Maxton et al. (1991)* | Y | N | Y | Y | N | N | Y | Y | 63 |
| *Melchor et al. (2020)* | Y | Y | Y | Y | Y | Y | Y | Y | 100 |
| *Soulier et al. (1996)* | Y | Y | Y | Y | N | N | Y | Y | 75 |
| *Vigren et al. (2013)* | N | N | Y | Y | N | N | N | Y | 38 |
| *%* | 75 | 50 | 100 | 88 | 25 | 13 | 63 | 75 | |

**Y = Yes, N = No, U = Unclear. Q1. Were the criteria for inclusion in the sample clearly defined? Q2. Were the study subjects and the setting described in detail? Q3. Was the exposure measured in a valid and reliable way? Q4. Were objective, standard criteria used for measurement of the condition? Q5. Were confounding factors identified? Q6. Were strategies to deal with confounding factors stated? Q7. Were the outcomes measured in a valid and reliable way? Q8. Was appropriate statistical analysis used?*

Appendix 4. Quality assessment of Case-Control Studies.

| *Citation* | Q1 | Q2 | Q3 | Q4 | Q5 | Q6 | Q7 | Q8 | Q9 | Q10 | Appraisal score (%) |
|---|---|---|---|---|---|---|---|---|---|---|---|
| *Barton et al. (1999)* | Y | Y | Y | Y | U | N | N | Y | N/A | Y | 60 |
| *Canataroğlu et al. (2001)* | U | U | U | Y | U | N | N | U | N/A | Y | 20 |
| *Lidèn et al. (2008)* | N | N | U | Y | U | N | N | U | N/A | Y | 20 |
| *Whorwell et al. (1986)* | Y | Y | Y | Y | Y | N | N | Y | N/A | Y | 70 |
| *Zimmerman (2003)* | Y | Y | Y | Y | Y | U | U | Y | N/A | Y | 70 |
| *%* | 60 | 60 | 60 | 100 | 40 | 0 | 0 | 60 | 0 | 100 | |

**Y = Yes, N = No, U = Unclear, N/A = Not applicable. Q1. Were the groups comparable other than the presence of disease in cases or the absence of disease in controls? Q2. Were cases and controls matched appropriately? Q3. Were the same criteria used for identification of cases and controls? Q4. Was exposure measured in a standard, valid and reliable way? Q5. Was exposure measured in the same way for cases and controls? Q6. Were confounding factors identified? Q7. Were strategies to deal with confounding factors stated? Q8. Were outcomes assessed in a standard, valid and reliable way for cases and controls? Q9. Was the exposure period of interest long enough to be meaningful? Q10. Was appropriate statistical analysis used?*

Appendix 5. Quality assessment of Cohort Studies.

| *Citation* | Q1 | Q2 | Q3 | Q4 | Q5 | Q6 | Q7 | Q8 | Q9 | Q10 | Q11 | Appraisal score (%) |
|---|---|---|---|---|---|---|---|---|---|---|---|---|
| *Mohammed et al. (2022)* | Y | U | Y | U | U | N | U | N | N | N | Y | 27 |
| *%* | 100 | 0 | 100 | 0 | 0 | 0 | 0 | 0 | 0 | 0 | 100 | |

**Y = Yes, N = No, U = Unclear. Q1. Were the two groups similar and recruited from the same population? Q2. Were the exposures measured similarly to assign people to both exposed and unexposed groups? Q3. Was the exposure measured in a valid and reliable way? Q4. Were confounding factors identified? Q5. Were strategies to deal with confounding factors stated? Q6. Were the groups/participants free of the outcome at the start of the study (or at the moment of exposure)? Q7. Were the outcomes measured in a valid and reliable way? Q8. Was the follow up time reported and sufficient to be long enough for outcomes to occur? Q9. Was follow up complete, and if not, were the reasons to loss to follow up described and explored? Q10. Were strategies to address incomplete follow up utilized? Q11. Was appropriate statistical analysis used?*

## Appendix 6. Quality assessment of Case Reports.

| *Citation* | Q1 | Q2 | Q3 | Q4 | Q5 | Q6 | Q7 | Q8 | Appraisal score (%) |
|---|---|---|---|---|---|---|---|---|---|
| *Barco et al. (2010)* | N | N | U | Y | Y | U | U | Y | 38 |
| *Cheung & Trudgill (2017)* | N | N | U | U | U | U | U | Y | 13 |
| *Widgren & MacGee (1990)* | N | N | U | U | U | U | U | Y | 13 |
| *%* | 0 | 0 | 0 | 33 | 33 | 0 | 0 | 100 | |

**Y = Yes, N = No, U = Unclear. Q1. Were patient's demographic characteristics clearly described? Q2. Was the patient's history clearly described and presented as a timeline? Q3. Was the current clinical condition of the patient on presentation clearly described? Q4. Were diagnostic tests or assessment methods and the results clearly described? Q5. Was the intervention(s) or treatment procedure(s) clearly described? Q6. Was the post-intervention clinical condition clearly described? Q7. Were adverse events (harms) or unanticipated events identified and described? Q8. Does the case report provide takeaway lessons?*
